# Suppression of Dendritic Cell Maturation by Kefir Peptides Alleviates Collagen-Induced Arthritis in Mice

**DOI:** 10.3389/fphar.2021.721594

**Published:** 2021-10-05

**Authors:** Chien-Fu Chen, Hsin-Pei Li, Ya-Hsuan Chao, Min-Yu Tu, Chih-Ching Yen, Ying-Wei Lan, Shang-Hsun Yang, Kowit-Yu Chong, Chi‐Chien Lin, Chuan-Mu Chen

**Affiliations:** ^1^ Department of Life Sciences, and Ph.D. Program in Translational Medicine, College of Life Sciences, National Chung Hsing University, Taichung, Taiwan; ^2^ Department of Orthopedic Surgery, Taichung Armed Forces General Hospital, Taichung, Taiwan; ^3^ Institute of Biomedical Sciences, National Chung Hsing University, Taichung, Taiwan; ^4^ Kaohsiung Armed Forces General Hospital Gangshan Branch, Kaohsiung, Taiwan; ^5^ Department of Health Business Administration, Meiho University, Pingtung, Taiwan; ^6^ Institute of Medical Science and Technology, National Sun Yat-Sen University, Kaohsiung, Taiwan; ^7^ Department of Internal Medicine, China Medical University Hospital, and College of Health Care, China Medical University, Taichung, Taiwan; ^8^ Department of Physiology, and Institute of Basic Medical Sciences, College of Medicine, National Cheng Kung University, Tainan, Taiwan; ^9^ Department of Medical Biotechnology and Laboratory Science and Graduate Institute of Biomedical Sciences, Division of Biotechnology, College of Medicine, Chang Gung University, Taoyuan, Taiwan; ^10^ Hyperbaric Oxygen Medical Research Lab, Bone and Joint Research Center, and Department of Laboratory Medicine, Chang Gung Memorial Hospital, Taoyuan, Taiwan; ^11^ The iEGG and Animal Biotechnology Center, and the Rong Hsing Research Center for Translational Medicine, National Chung Hsing University, Taichung, Taiwan

**Keywords:** kefir peptides, rheumatoid arthritis, collagen-induced arthritis, bone marrow-derived dendritic cells, CD4^+^ T cells

## Abstract

Arthritis is a disorder that is characterized by joint inflammation and other symptoms. Rheumatoid arthritis (RA), an autoimmune disease, is one of the most common arthritis in worldwide. Inflammation of the synovium is the main factor that triggers bone erosion in the joints in RA, but the pathogenesis of RA is not clearly understood. Kefir grain-fermented products have been demonstrated to enhance immune function and exhibit immune-modulating bioactivities. This study aims to explore the role of kefir peptides (KPs) on the regulation of dendritic cell, which are found in RA synovial fluid, and the protection effects of KPs on mice with collagen-induced arthritis (CIA). Immature mouse bone marrow-derived dendritic cells (BMDCs) were treated with KPs (2.2 and 4.4 mg/ml) and then exposed to lipopolysaccharide (LPS) to study the immune regulation function of KPs in dendritic cells. Mice with CIA (n = 5 per group) were orally administrated KPs (3.75 and 7.5 mg/day/kg) for 21 days and therapeutic effect of KPs on mice with arthritis were assessed. In this study, we found that KPs could inhibit surface molecule expression, reduce inflammatory cytokine release, and repress NF-κB and MAPK signaling in LPS-stimulated mouse BMDCs. In addition, a high dose of KPs (7.5 mg/kg) significantly alleviated arthritis symptoms, decreased inflammatory cytokine expression, suppressed splenic DC maturation and decrease the percentage of Th1 and Th17 in the spleens on mice with CIA. Our findings demonstrated that KPs ameliorate CIA in mice through the mechanism of suppressing DC maturation and inflammatory cytokine releases.

## Introduction

Arthritis is a chronic disorder that involves joint inflammation, and the related symptoms generally include joint pain and stiffness. Two of the most common types of arthritis are osteoarthritis (OA) and rheumatoid arthritis (RA) (Kidd et al., 2007). OA is a painful condition caused by gradually mechanical wear and tear on joints, whereas RA is an autoimmune disease arising from an abnormal immune response in which patient’s own immune system attacks the joints (Parekh et al., 1985). The pathogenesis of RA is not clearly understood but may involve genomic variations, gene transcription, protein translation and posttranslational modifications (Song and Lin, 2017). The erosion of cartilage and bone is a classical feature of RA. One of the major factors that triggers cartilage and bone erosion in joints during RA progression is inflammation of the synovium (Chabaud et al., 2000; Won et al., 2011).

Previous studies demonstrated the importance of activated T cells in RA pathogenesis. The collagen-induced arthritis (CIA) animal model demonstrated the involvement of activated pro-inflammatory Th1 and Th17 cells, as well as suppressed Treg cells may be involved in the pathogenesis of RA (Sarkar et al., 2010). In addition, dendritic cells (DCs), also known as sensory cells, are antigen-presenting cells (APCs) that display antigen complexes in the context of major histocompatibility complexes (MHCs) on the cell surfaces to induce immune responses (Kambayashi and Laufer, 2014). DCs are derived from bone marrow stem cells (BMSC) and found in an immature state in the blood. DCs mainly function to process antigens, upregulate costimulatory molecule expressions and migrate to lymph nodes that polarize naïve T-cell differentiation into, such as Th1, Th2 or Th17 effector cells to enhance immunity against foreign antigens and tolerance to self-antigens during activation. DCs also secrete many important cytokines to adjust immune responses (Blanco et al., 2008; Chang et al., 2014). However, both immature and mature DCs have been found to accumulate in the synovial joint tissues in RA patients (Pettit et al., 2000; Lebre et al., 2008).

The excessive production of pro-inflammatory cytokines or chemokines and the continuous presentation of autoantigens by DCs in the CIA mice are considered to be the important factors leading to the pathogenesis and progression of arthritis (Leung et al., 2002). Therefore, inhibiting the up-regulation of DCs is a potential strategy to regulate immune-mediated rheumatic diseases (Khan et al., 2009; Benham et al., 2015; Ahmed and Bae, 2016). Recent researches seeking to inhibit the maturation of DCs in RA has attracted great attention to the development and exploration of RA drugs. For example, gold sodium thiomalate and leflunomide derivatives have recently been shown to affect the maturation and differentiation of DCs, and may reduce the progression of RA (Wang et al., 2002; Kirsch et al., 2005; Zeyda et al., 2005).

Kefir is produced from milk proteins by a microbial community derived from fermented kefir grains (Tu et al., 2015). The health benefits of kefir products have mostly been elucidated; these benefits include improved gastrointestinal flora, increased immune modulatory, antithrombotic, antimicrobial and calcium absorption bioactivities (Chen et al., 2020). Our previous studies demonstrated that treatment with KPs decreased PM_4.0_-induced reactive oxygen species (ROS) generation, significantly reduced p-NF-κB, NLRP3, caspase-1, IL-1β, IL-4, IL-6, and TNF-α expression and also increased SOD antioxidant activity in an NF-κB-Luc^+/+^ transgenic mouse model (Chen et al., 2019).

In this study, we focused on the investigation of crucial role of KPs in the regulation of immune function in BMDCs *in vitro*, and in a mouse model of CIA *in vivo*, which is the most commonly utilized model for autoimmune RA.

## Materials and Methods

### Ethics Statement

The animal experiments were approved by the Institutional Animal Care and Use Committee of National Chung Hsing University, Taiwan (IACUC no. 109-106) and were carried out in compliance with institutional guidelines.

### Animals

Female DBA/1 mouse strain (aged 6–8 weeks) was obtained from the Jackson Laboratory (Bar Harbor, ME, United States), and OT-II TCR transgenic mice were kindly provided by Prof. Ching-Liang Chu (National Taiwan University, Taipei, Taiwan). All the mice were housed under controlled conditions with ad *libitum* supply of chow and water in a specific pathogen-free (SPF) animal facility.

### Preparation of KPs

Kefir starter grains purchased from Phermpep Co. (Taichung, Taiwan) were inoculated in sterilized fresh milk and incubated at 20°C for overnight to activate the grains. The grains were retrieved with a fine-mesh sieve, reinoculated into sterilized milk protein broth and incubated at the condition as previously described (Shi et al., 2018; Tu et al., 2020). The total peptide content in the KPs powder, calculated as the triglycine equivalent, was 23.1 g/100 g.

### Preparation of Bone Marrow-Derived DCs

BMDCs were isolated from mouse bone marrow cells as previously described (Chen et al., 2016; Yang et al., 2020). Briefly, the bone marrow cells were flushed from the tibiae and femurs of female DBA/1 or OT-II mice, and the cells were cultured in RPMI-1640 medium (Thermo Fisher Scientific, Waltham, MA, United States). On days 2, 4 and 6 of culture, the nonadherent cells were gently removed, and replaced with fresh medium supplemented with GM-CSF (20 ng/ml) and IL-4 (10 ng/ml). The loosely adherent cells were harvested on day 7 and considered immature BMDCs.

### Flow Cytometry Analysis of Surface Maker Expression by BMDCs and Splenic DCs

For detection of BMDC differentiation *in vitro*, the 1:1000 dilution of anti-CD11c antibody was utilized to detect the expression of CD11c marker via an Accuri five flow cytometer following 2.2 or 4.4 mg/ml of KPs were added to the culture media. Fresh KPs were supplemented each time whenever the medium was changed. BMDCs with the density of 1 × 10^6^ cells/well were pretreated with KPs (2.2 or 4.4 mg/ml) for 1 h and then stimulated by LPS (100 ng/ml) for 24 h. After treatment, the cells were stained at 4°C for 30 min with a FITC-labeled mouse anti-CD11c antibody, PE-labeled anti-CD40, CD80 and CD86 antibodies or isotype-matched control antibody, and the cells were subsequently analyzed by an Accuri five flow cytometer (BD Biosciences, Franklin Lakes, NJ, United States). The mean fluorescence intensity (MFI) of surface markers, CD40, CD80 and CD86, were calculated by Accuri C6 software (BD Biosciences) after gating based on forward scatter (FSC) and CD11c^+^ expression. Splenocytes were prepared from the spleen on day 42, and a single cell suspension was obtained. The cellular markers expressed by the splenocytes were also analyzed by the Accuri five flow cytometer after gating based on FSC and CD11c^+^ expression to identify splenic dendritic cells.

### CCK-8 Cell Viability Assay

The viability of BMDCs was determined by a CCK-8 colorimetric assay accordingly (Sigma-Aldrich, St. Louis, MO, United States). Briefly, BMDCs (5×10^4^ cells/well) were plated on 96-well plates, incubated in the presence or absence of KPs (2.2 or 4.4 mg/ml) for 1  h, and stimulated with LPS (100 ng/ml) for 24 h. After treatment, CCK-8 solution (10 µl) was added to the test wells and incubated for 4 h. Then, the O.D. absorbance was detected at a wavelength of 450 nm.

### Lactate Dehydrogenase Assay

Cellular toxicity was examined based on LDH release from cells into the culture medium using an LDH assay kit (Cayman Chemical Co., Ann Arbor, MI, United States). The amount of LDH was measured at a wavelength of 450 nm with a microplate reader (Tecan, Durham, NC, United States).

### Cytokine Measurement *in vitro*

BMDCs (5×10^4^ cells/well) were incubated in the presence or absence of KPs (4.4 mg/ml) for 1 h, and stimulated with 100 ng/ml LPS, 20 μg/ml zymosan, 20 μg/ml lipoteichoic acid, 250 μg/ml polyinosine polycytidylic acid, 500 ng/ml flagellin, 20 ng/ml Pam2Cys-Ser-Lys4, or 200 nM CpG oligodeoxynucleotides-1826 for 24 h. The cytokine levels of IL-6, IL-12p70, IL-23 and TNF-α were quantified in cell-free supernatants using mouse ELISA kits (Thermo Fisher Scientific).

### OVA-specific T Cell Activation

BMDCs were purified from OT-II mice with magnetic beads conjugated to anti-CD11c mAb (Miltenyi Biotec, Auburn, CA, United States) and were pulsed with 2 μg/ml OVAP2 (Echo Chemical Co., Taichung, Taiwan) and incubated with LPS (100 ng/ml), KPs (4.4 mg/ml), or LPS + KPs for 18 h. After incubation, the BMDCs (5 × 10^5^) were cocultured with OVAP2-specific CD4^+^ T cells that were negatively enriched from the spleens of OT-II mice at cellular ratios of 1:5 (CD11c^+^ DC:CD4^+^ T cell) for 72 h ^3^H-labeled thymidine (1 μCi/well) was added for the last 18 h (Tian et al., 2020). The cells were collected, and the ^3^H-thymidine incorporation was quantified by liquid scintillation counting (Beckman, Boulevard Brea, CA, United States).

### Western Blotting Analysis

Purified BMDCs were pretreated with or without KPs (2.2 or 4.4 mg/ml) for 1 h and further incubated for 6 h with 100 ng/ml LPS before being harvested. The protein blots were incubated overnight at 4°C with primary antibodies against p38, phospho-p38 (Thr180/Tyr182), p42/44 (137F5), phospho-p42/44 (Thr202/Tyr204, 20G11), JNK, phospho-JNK (81E11), or GAPDH. The appropriate horseradish peroxidase (HRP)-labeled secondary antibodies (Jackson ImmunoResearch, West Grove, PA, United States) were used. The bands were developed with enhanced chemiluminescence (ECL) detection kit reagent (GE Healthcare Life Sciences). All the band densities were quantified by densitometric analysis using ImageJ 1.47 software (NIH, Bethesda, MD, United States) and normalized to the expression of GAPDH in each lane (Tung et al., 2020).

### Nuclear Extract Preparation and NF-κB/p65 Activity Assay

BMDCs were pretreated with or without KPs (4.4 mg/ml) for 1 h and further cultured for 6 h with LPS (100 ng/ml) before being harvested. A NE-PER extraction system (Thermo Fisher Scientific) was used to prepare the nuclear extracts. To detect nuclear NF-κB/p65 binding, a total of 20 μg nuclear extract was used in a Trans-AM NF-κB ELISA kit (Active Motif North America, Carlsbad, CA, United States).

### Th1 and Th17 Cell Differentiation *in vitro*

Purified CD4^+^ T cells were obtained from the spleens of naïve DBA/1 mice and stimulated with 1 μg/ml plate-coated anti-CD3 (clone 2C11) and 1 μg/ml soluble-form anti-CD28 (clone PV1.17) under Th1-polarizing conditions [5 ng/ml IL-2, 10 ng/ml IL-12 and 2 μg/ml anti-IL-4 antibody (Biolegend, San Diego, CA, United States)] or Th17-polarizing conditions (20 ng/ml IL-6, 2.5 ng/ml TGF-β, 10 μg/ml anti-IFN-γ and 10 μg/ml anti-IL-4), and the cells treated with or without KPs (4.4 mg/ml) starting at the beginning of the induction. The cells were collected on day 4 of KPs treatment, and intracellular cytokine analysis was performed.

### Induction of CIA and KPs Treatment

Induction of arthritis was achieved using an established method (Brand et al., 2007). Briefly, CIA was induced by a single subcutaneous injection of 100 µg bovine type II collagen (CII; Morwell Diagnostic, Zurich, Switzerland) emulsified with equal Freund’s complete adjuvant (Difco Laboratories, Franklin Lakes, NJ, United States) into the base of the tail. On day 21, all the mice were boosted with an injection of 100 µg CII emulsified in Freund’s incomplete adjuvant (Difco Laboratories). After the CII induction of arthritis, the mice were randomly distributed into four groups as follows: 1) no immunization (normal; n = 5); 2) CIA control (n = 5); 3) CIA treated with a high dose of KPs (7.5 mg/day/kg body weight (BW), n = 5); and 4) CIA treated with a low dose of KPs (3.75 mg/day/kg BW, n = 5). The different doses of KPs were suspended in 10 ml ddH_2_O and orally administered at the same volume of 0.2 ml per mouse every day for 3 weeks.

### Clinical Assessment of Arthritis

The arthritis score for clinical assessment was evaluated in each paw and recorded every 3 days using a scoring system as previously described (Yue et al., 2017). After booster immunization, the mouse paws were assessed using a 0–4 points scale based on increasing levels of paw swelling, erythema, edema and joint rigidity. The arthritis score for each mouse was the sum of the severity of all four paws (maximum 16 points for each mouse).

### Histological Evaluation

After the mice was sacrificed (on day 42), the right hind limbs were harvested and fixed in 10% formalin. The sections were stained with hematoxylin and eosin (H&E) and observed under light microscopy at a magnification of 100 × (Lan et al., 2019; Chen et al., 2020). Histological changes were scored in the parameters as previously described (Wasserman, 2011): 1) cell infiltration, 2) synovial hyperplasia and 3) cartilage destruction. Scores of 0–3 were assigned on the following criteria: 0, no changes; 1, mild changes; 2, moderate changes; and 3, severe changes.

### Cytokine Measurement in Paw Tissues

Frozen paw tissues (100 mg) were homogenized in RIPA lysis buffer (1 ml) containing a protease inhibitor cocktail (Pierce, Rockford, IL, United States), and the protein concentration was calculated with a bicinchoninic acid (BCA) assay kit (Thermo Fisher Scientific). The levels of the cytokines, IFN-γ, IL-6, IL-10, IL-17A, and TNF-α, were measured using murine ELISA kits (eBioscience Inc., San Diego, CA, United States) (Yen et al., 2020).

### Analysis of Anti-Collagen Type II (CII) IgG Antibodies

Blood samples were collected on day 42. The anti-CII IgG antibody titers were analyzed with commercially available kits and anti-mouse CII IgG TMB (2036T; Chondrex, Redmond, WA, United States) according to the manufacturer’s instructions.

### Spleen Cell Proliferation and Cytokine Production in Response to Collagen II

Splenocytes (2 × 10^6^ cells/ml) were cultured in 200 μl of medium containing fetal calf serum (10%), gentamicin (50 μg/ml), glutamine (2 mM), and 2-mercaptoethanol (50 μM). The splenocytes were stimulated with 20 μg/ml mouse CII for 72 h ^3^H-labeled thymidine incorporation was quantified to assess the cellular proliferation. The numbers of CD4^+^IFN-γ^+^ Th1 cells and CD4^+^IL-17A^+^ Th17 cells were analyzed to assess intracellular cytokine levels.

### Statistical Analysis

All the data are presented as the mean ± SEM. One-way or two-way ANOVA followed by Dunnett’s multiple comparison test was used to compare multiple experimental groups with GraphPad Prism v5.0 (La Jolla, CA, United States). A *p* ≤ 0.05 was considered statistically significant.

## Results

### KPs Inhibit Surface Costimulatory Molecules in LPS-Stimulated Mouse BMDCs

**FIGURE 1:**
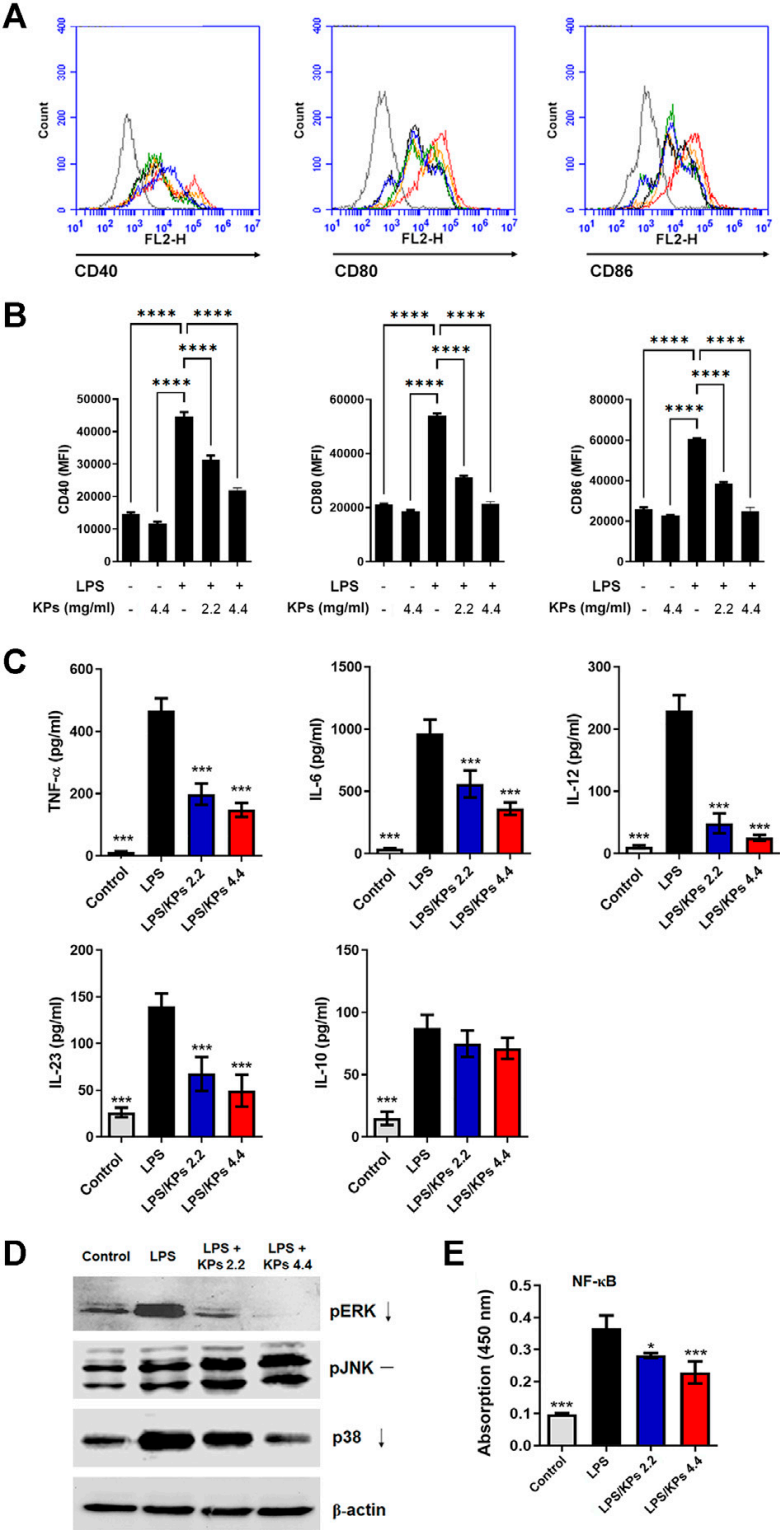
Effect of KPs on the expression of immunomodulatory cell surface markers, the cytokine release, MAPK phosphorylation and NF-κB translocation in LPS-treated mouse BMDCs. Immature murine BMDCs were stimulated with LPS (100 ng/ml) in the presence or absence of KPs (2.2 mg/ml and 4.4 mg/ml) for 24 h. **(A)** Representative histogram graphs of the mean fluorescence intensity (MFI) of costimulatory molecules CD40, CD80 and CD86 on CD11c^+^ DC cells examined by flow cytometry. **(B)** MFI values presented as the mean ± SEM from three independent experiments (n = 3). ****p < 0.0001, as determined by one-way ANOVA test. **(C)** Cell-free supernatants were collected, and cytokine production was measured by ELISA. The concentrations are presented as the mean ± SEM (n = 3). **(D)** Immature BMDCs were stimulated with LPS (100 ng/ml) with or without KPs (2.2 mg/ml and 4.4 mg/ml) and lysed at 6 h after treatment. The levels of phosphorylated pERK, pJNK and p38 MAPK in whole cell lysates were analyzed by Western blotting. Representative images from one of three independent experiments are shown. **(E)** The concentration of NF-κB/p65 in nuclear extracts was measured using a Trans^AM^ assay. The values are presented as the mean ± SEM (n = 3). *p < 0.05; ***p < 0.001 vs the LPS alone control group, as determined by one-way ANOVA with Dunnett’s test.

Immature mouse BMDCs were treated with KPs (2.2 and 4.4 mg/ml) and then exposed to LPS which is a bacterial product known to induce DC maturation. The LPS-stimulated mouse BMDCs significantly increased the expression levels of the surface costimulatory molecules, CD40, CD80 and CD86, while KPs treatment reduced the expression of these molecules in a dose-dependent manner (Figures 1A,B, and also Supplementary Figure S1). Data showed that KP-treated alone at the high concentration (4.4 mg/ml) did not cause a notable change in immature mice BMDCs compared to vehicle control. Furthermore, these effects did not occur due to cytotoxicity because we used LDH and CCK-8 (Supplementary Figure S2A, B, respectively) assays to confirm that the cell viability in the KPs-treated groups was not significantly different from that in the water-treated control group.

### KPs Suppress Cytokine Release From LPS-Stimulated Mouse BMDCs

As shown in Figure 1C, KPs-pretreated mouse BMDCs secreted lower concentrations of proinflammatory cytokines, including TNF-α, a Th1-specific cytokine (IL-12) and Th17-specific cytokines (IL-6 and IL-23), following LPS stimulation. However, KPs did not significantly affect the anti-inflammatory cytokine IL-10 expression in LPS-stimulated BMDCs. Moreover, we stimulated BMDCs with other TLR ligands, including zymosan, lipoteichoic acid, synthetic diacylated lipoprotein Pam2Cys-Ser-Lys4, peptidoglycan (TLR1, 2, or six ligands), polyinosine polycytidylic acid (TLR3 ligand), flagellin (TLR5 ligand), and synthetic CpG oligodeoxynucleotides (TLR9 ligand). Results showed that KPs (4.4 mg/ml) significantly reduced TNF-α expression level in response to all these ligands (Supplementary Figure S3).

### KPs Suppress NF-κB and MAPK Pathways in LPS-Stimulated BMDCs

To discover the molecular mechanisms underlying the inhibitory effect of KPs, we determined the effect of KPs on MAPK and NF-κB activation in LPS-stimulated BMDCs. As shown in Figure 1D, the levels of phosphorylated MAPKs, namely, ERK, p38, and JNK, were significantly increased in the LPS-stimulated BMDC group relative to the control BMDC group. In contrast, the phospho-ERK and p38 levels were greatly decreased in the KPs treatment group (also shown in Supplementary Figure S4 for the original blots). Furthermore, we used Trans^AM^ assays to measure the concentrations of NF-κB/p65 in nuclear extracts. The nuclear translocation of NF-κB/p65 was significantly increased in BMDCs after stimulation with LPS. However, pretreatment with KPs significantly reduced this effect in a dose-dependent manner (Figure 1E).

### KPs Inhibit the Ability of Mouse BMDCs to Trigger CD4^+^ T Cell Proliferation and Th1/Th17 Cytokine Production in Mixed Cell Culture Reactions

**FIGURE 2:**
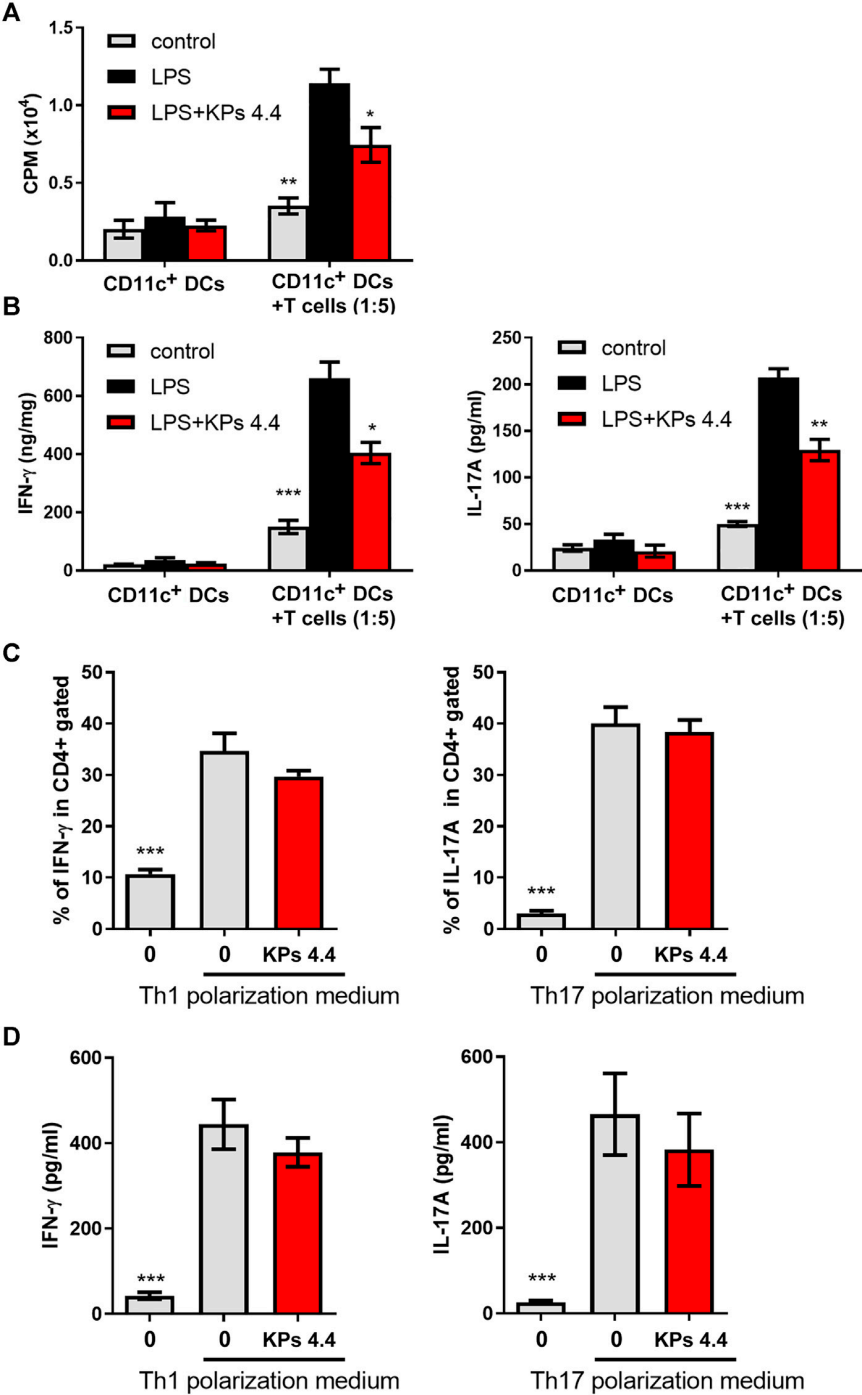
Effects of KPs on CD4^+^ T cells proliferation, LPS-stimulated BMDC cytokine production *in vitro*, and Th1 and Th17 differentiation *in vitro*. **(A)** OT-II-specific CD4^+^ T cells were cocultured with BMDCs in the presence of OVA and LPS. KPs were added at a concentration of 4.4 mg/ml at the beginning of the induction. After 3 days of culture, the changes in T cell proliferation via the ^3^H-TdR incorporation assay were quantified in counts per minute (CPM) by liquid scintillation counting. **(B)** The levels of IFN-γ and IL1-7A in the supernatants of the cocultures. The values are presented as the mean ± SEM (n = 3). *p < 0.05; **p < 0.01; ***p < 0.001 vs the LPS alone group, as determined by one-way ANOVA with Dunnett’s test. **(C)** Naïve CD4^+^ T cells were cultured in Th1- or Th17-polarizing conditioned medium. KPs were added at a concentration of 4.4 mg/ml at the beginning of induction. Bar graphs showing the percentages of CD4^+^ T cells expressing IFN-γ and IL-17A, as determined by flow cytometry. **(D)** The levels of IFN-γ and IL-17A produced under Th1- or Th17-polarizing conditions were examined with ELISA kits. The data are expressed as the mean ± SEM (n = 3). ***p < 0.001 compared to the LPS-stimulated group without KPs treatment, as determined by one-way ANOVA with Dunnett’s test.

To elucidate the relevance of the KPs-mediated suppression of DC functions to immune responses, we analyzed the effects of KPs in a coculture system that induced ovalbumin (OVA)-specific CD4^+^ T cells from OT-II transgenic mice and BMDCs treated under different conditions. The proliferation results in Figure 2A showed that coculture with LPS-stimulated BMDCs effectively increased proliferative responses, while LPS-stimulated DCs were pretreated with KPs, a reduced amount of [^3^H] uptake was observed. In addition, the ELISA results revealed decreased Th1 (IFN-γ) and Th17 (IL-17A) cytokine production in the cocultures that included KPs-treated BMDCs (Figure 2B).

### KPs Do Not Directly Affect Th1 and Th17 Cell-Lineage Differentiation

We next extended to elucidate whether KPs could directly affect T cell lineage differentiation. As shown in Figures 2C,D, the administration of KPs to mouse naïve CD4^+^ T cells cultured under Th1- or Th17-polarizing conditions did not significantly affect the numbers of IFN-γ- and IL-17A-producing cells.

### KPs Alleviate the Symptoms of CIA in DBA/1 Mice

**FIGURE 3:**
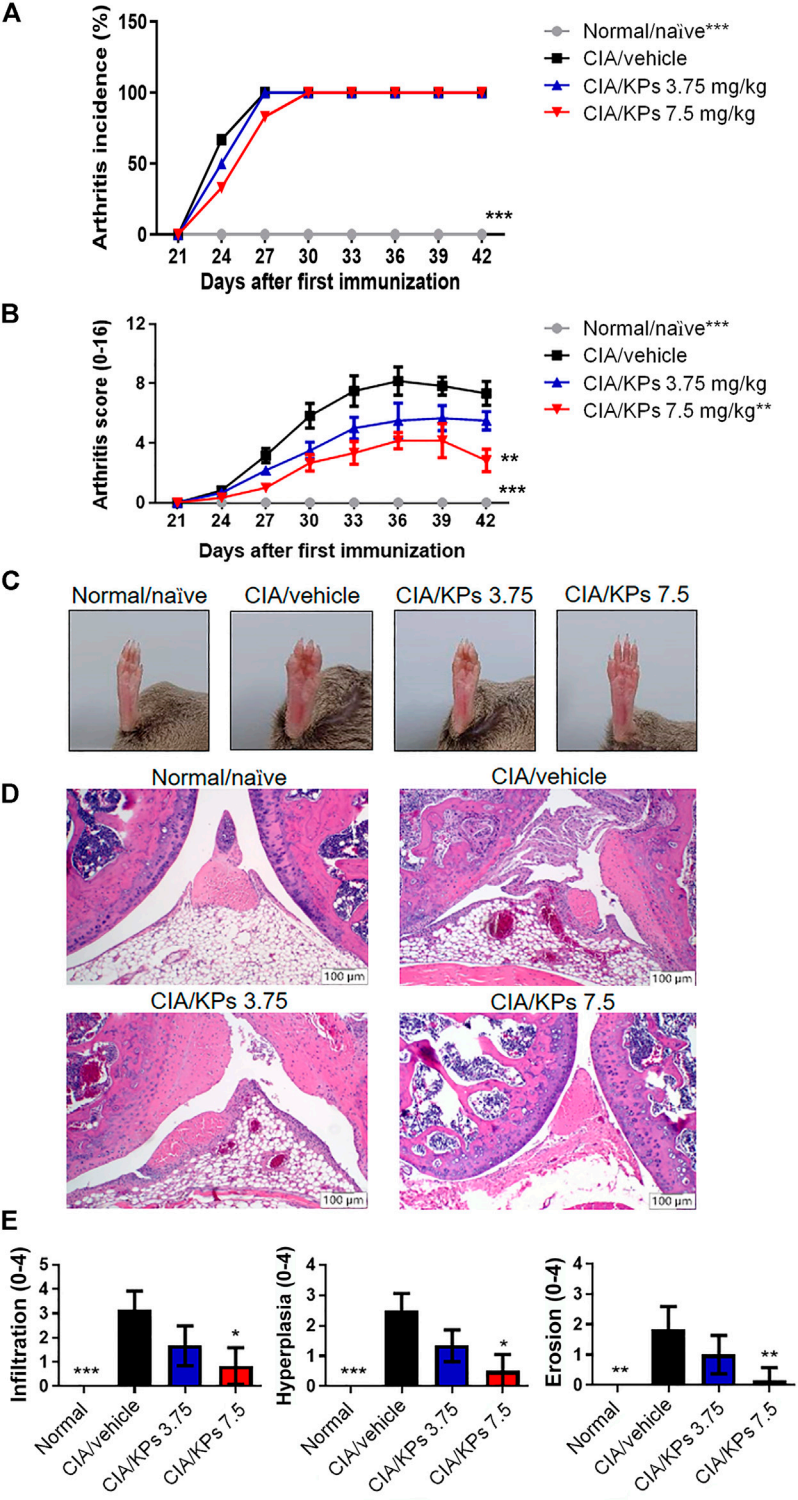
Effects of KPs on type II CIA severity. The arthritis incidence **(A)** and arthritis score **(B)** were observed every 3 days after type II collagen (CII) booster immunization (day 21). **(C)** The whole view of the severity of arthritis in the right legs of the mice. Characterization of paw swelling, erythema, edema, and joint rigidity in groups subjected to different treatment. **(D)** Representative photographs of histopathological examination by H&E staining (100× magnification) on day 42. **(E)** Pathogenic scores of CIA were determined. The data are presented as the mean ± SEM (n = 5). *p < 0.05; **p < 0.01; ***p < 0.001 vs the CIA/vehicle control group, as determined by two-way ANOVA (**A**, **B**) or one-way ANOVA with Dunnett’s test **(E)**.

In the typical arthritic animal model, CIA mice, the excessive production of pro-inflammatory cytokines or chemokines and continuous presentation of autoantigens by DCs are regarded as contributing factors to the pathogenesis and progression of murine arthritis (Leung et al., 2002). Based on the *in vitro* finding that KPs sufficiently suppressed BMDCs maturation and function, we further investigate the immunosuppressive properties of KPs in the murine CIA model. Although KP cannot effectively reduce the incidence of CIA arthritis (Figure 3A), but the administration of a high dose of KPs (7.5 mg/kg), starting at day 1 after the second immunization, significantly ameliorated the severity of CIA, which was characterized by paw swelling, erythema and joint rigidity (Figures 3B,C). In addition, H&E staining was performed to evaluate the histopathological changes in the joints (Figure 3D). KPs administration significantly reduced synovial tissue inflammatory infiltration, hyperplasia, and cartilage erosion in the knee joints in a dose-dependent manner (Figure 3E).

### KPs Decrease the Production of Inflammatory Cytokines in the Paws of Mice With CIA

**FIGURE 4:**
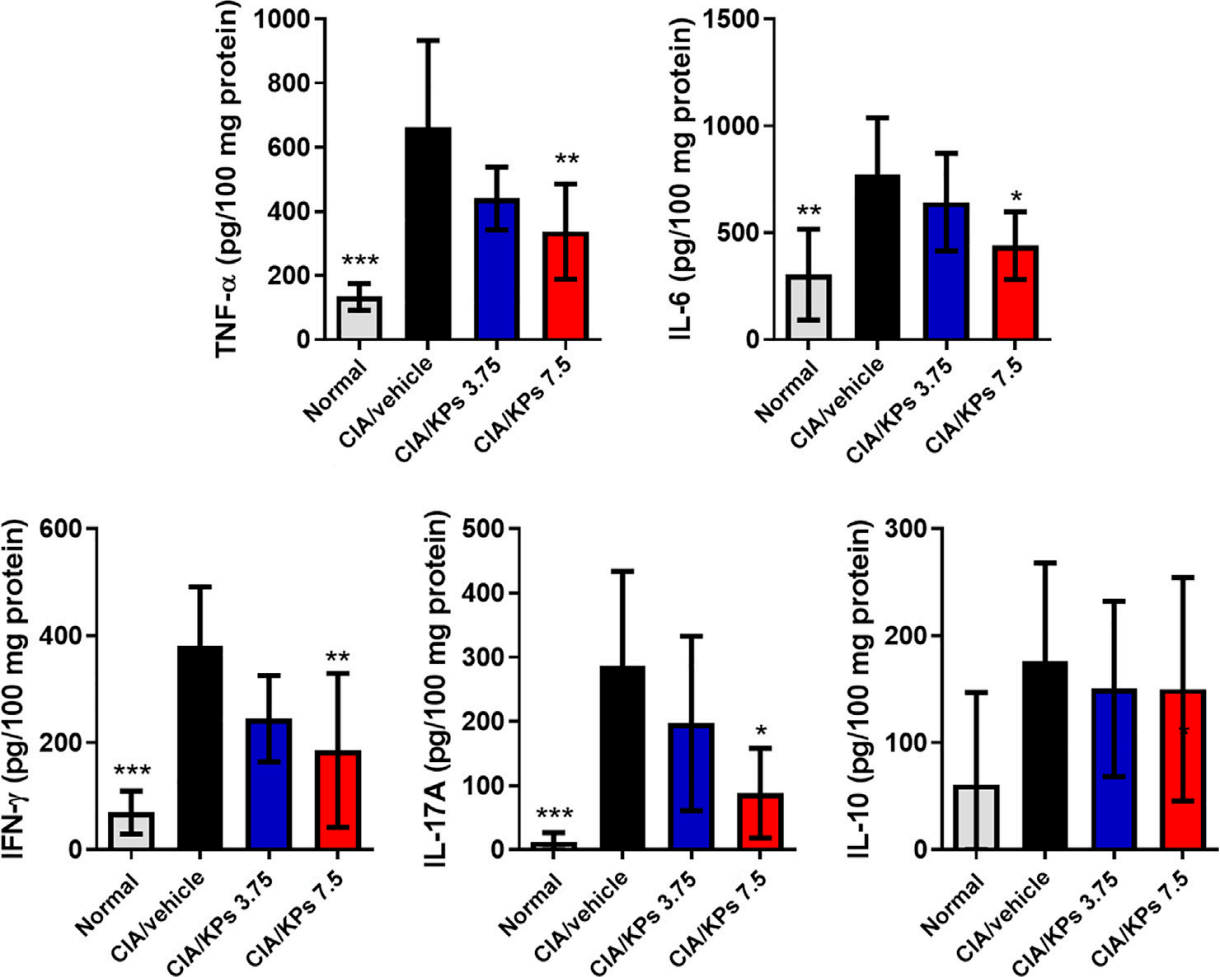
Effects of KPs on proinflammtory cytokine expression in the paws of CIA mice. The paw tissue homogenates were obtained on day 42. The cytokines TNF-α, IL-6, IFN-γ, IL-17A, and IL-10 were measured by ELISA. The data are presented as the mean ± SEM (n = 5). *p < 0.05; **p < 0.01; ***p < 0.001 vs the CIA/vehicle control group, as determined by one-way ANOVA with Dunnett’s test.

The ELISA results revealed remarkable expression of the inflammatory cytokines, including TNF-α, IL-6, IFN-γ and IL-17A, in the paws of CIA/vehicle control mice (Figure 4). In contrast, the expression levels of these cytokines were significantly reduced after KPs treatment, especially after treatment with the high dose of KPs (7.5 mg/kg). On the contrary, the anti-inflammatory cytokine, IL-10, showed no significant changes regardless of KPs dose (Figure 4).

### KPs Suppress Collagen-specific Immune Responses in Mice With CIA

**FIGURE 5:**
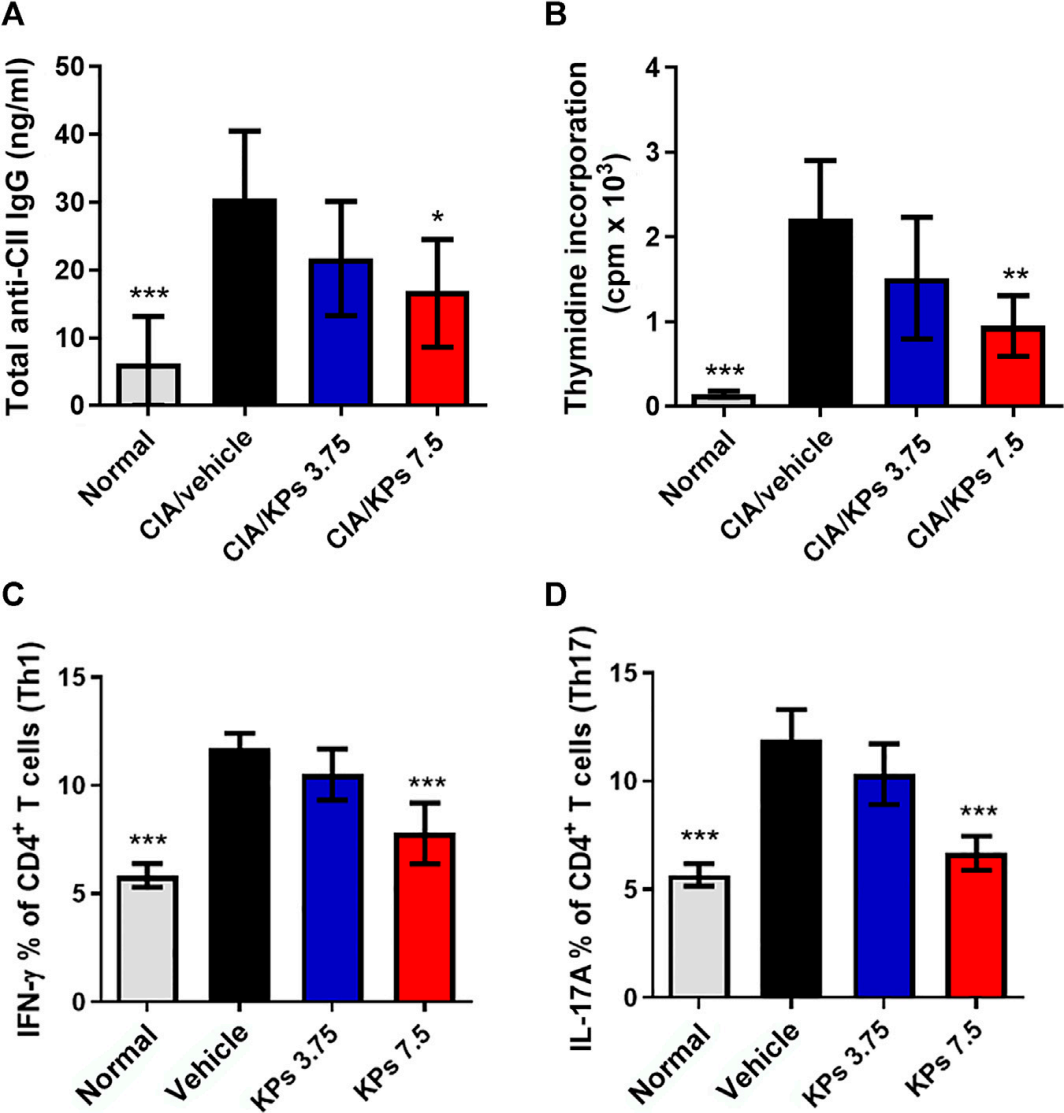
Effect of KPs on CII-specific immune responses in serum and the CD4^+^ T cell subset profiles in the spleens of CIA mice. **(A)** Sera were collected by orbital sinus venipuncture at day 42 after primary immunization to estimate anti-type II collagen (IgG) antibody levels by ELISA. **(B)** Splenocytes were harvested at day 42. Cultures were pulsed with ^3^H-thymidine (1 μCi/well) and the incorporation of radioactivity was quantified in counts per minute (cpm) by liquid scintillation counting after 18 h of incubation. Intracellular flow cytometry analysis of cells collected from the spleens of the mice in different groups on day 42 after immunization. Flow cytometry analysis of Th1 and Th17 cells. The cells were gated on the CD4^+^ population. Bar graph of the quantification of CD4^+^ T cell subsets, Th1 **(C)** and Th17 **(D)**, plotted from three independent experiments. The data are expressed as the mean ± SEM (n = 5). *p < 0.05; **p < 0.01; ***p < 0.001 vs the CIA/vehicle control group, as determined by one-way ANOVA with Dunnett’s test.

We further estimated the anti-type II collagen antibody (anti-CII IgG) titers in cultures of serum and spleen cells that proliferated in response to CII. The results showed that suppression of the anti-CII IgG antibody titer in the KPs-treated CIA groups (Figure 5A). In addition, the results in Figure 5B revealed that CII significantly increased the spleen cell proliferation in the CIA/vehicle group compared to the normal control group (*p* < 0.001). Nevertheless, KPs oral administration significantly decreased the CII-induced spleen cell proliferation in the CIA mouse groups in a dose-dependent manner (Figure 5B).

### KPs Decrease the Ratio of Th1 and Th17 Cells in the Spleen Tissues of Mice With CIA

Subsequently, we assessed the CD4^+^ T cell subset profiles in the lymphocytes from the spleens of mice with CIA subjected to different treatments. Compared with that in the naïve mouse group, the ratio of splenic CD4^+^IFN-γ^+^ Th1 cells (Figure 5C) and CD4^+^IL-17A^+^ Th17 cells (Figure 5D) in the CIA/vehicle control group was significantly higher, and the percentages of Th1 and Th17 cells were significantly decreased in groups of mice with CIA treated with the high dose of KPs (*p* < 0.001).

### KPs Suppress Splenic DC Maturation in Mice With CIA

**FIGURE 6:**
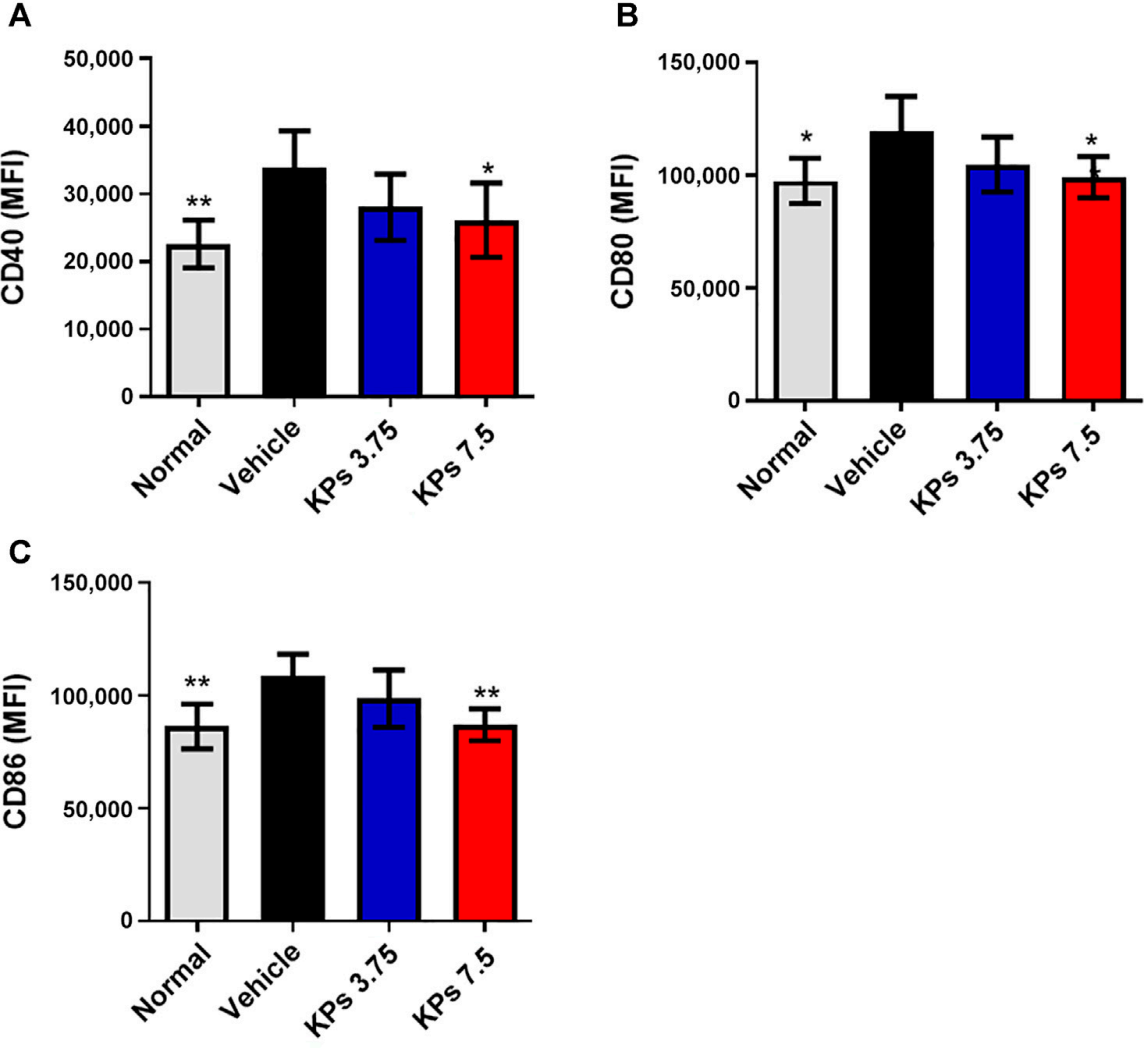
Effect of KPs on splenic DC maturation in mice with CIA. Flow cytometry analysis of cells collected from the spleens of the mice in different groups on day 42 after immunization. MFI of CD40 **(A)**, CD80 **(B)**, and CD86 **(C)** on CD11c^+^ DC cells was examined by flow cytometry. Bar graphs show the quantified data from three independent experiments. The values are presented as the mean ± SEM (n = 5). *p < 0.05; **p < 0.01; ***p < 0.001 vs the CIA/vehicle control group, as determined by one-way ANOVA with Dunnett’s test.

To understand whether KPs can also affect the maturation of dendritic cells in the CIA model, we further analyzed the expression of the costimulatory molecules, including CD40, CD80 and CD86, in splenic dendritic cells using flow cytometry (Supplementary Figures S5, 6). Results showed that despite the elevation in surface marker expression, high-dose KPs (7.5 mg/kg/day) treatment was sufficient to suppress the expression of the costimulatory molecules, CD40 (Figure 6A), CD80 (Figure 6B) and CD86 (Figure 6C), by the splenic DCs of mice with CIA.

### The Effect of KPs on BMDC Differentiation

**FIGURE 7:**
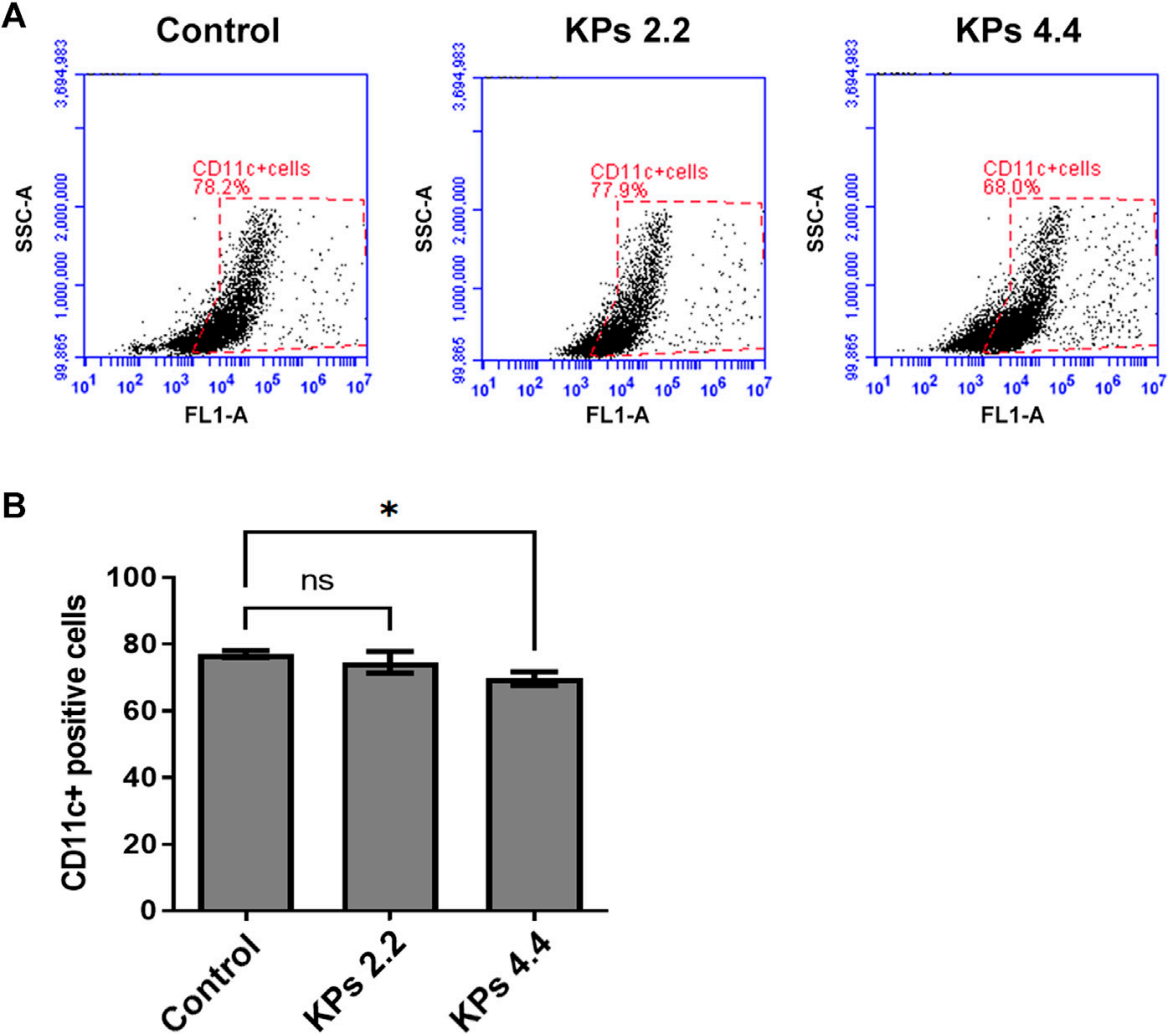
Effect of KPs on differentiation of CD11c^+^ BMDCs. **(A)** Flow cytometry analysis of the bivariate graphs of BMDCs of CIA mice cultured with the indicated concentration of KPs followed by evaluation of CD11c^+^ expressions. **(B)** The mean ± SD of the experimental triplicates are presented in the bar graph. Experiments were repeated three times independently. ***p < 0.001 vs 0.1% DMSO-treated mock group, as determined by a one-way ANOVA with Dunnett’s test.

Given there was only a small amount of DCs can be retrieved from peripheral blood, synovial tissues, and spleens in mice, we performed an *in vitro* study to elucidate whether DC differentiation was affected by KPs (Figure 7). To this end, we isolated myeloid cells from CIA murine bone marrow-derived cells and added KPs (2.2 or 4.4 mg/ml) into the culture medium to incubate and investigate the proportions of CD11c^+^ cells within the myeloid cells (Figure 7A). Compared with the DMSO control, the percentage of CD11c^+^ cells were significantly reduced at high doses of KP (4.4 mg/ml), but not at low dose of KP (2.2 mg/ml) (Figure 7B), suggesting that the effect of KPs on DC *in vivo* is still mainly in its effect of inhibiting maturation.

## Discussion

RA is an autoimmune disorder that usually occurs with age and affects the hands and feet (Tian et al., 2020). Although the pathogenesis of RA is still not fully understood, substantial insight into the cellular and molecular mechanisms involved in RA pathogenesis has been obtained in the past decade (Wasserman, 2011; Yue et al., 2017). Bone and cartilage erosions are the major feature of RA (Lee and Weinblatt, 2001). Moreover, the main factor that triggers bone and cartilage erosions in the joints during RA is inflammation of the synovium (Marok et al., 1996; Ideguchi et al., 2006; Schett and Gravallese, 2012; Kim et al., 2015; Komatsu and Takayanagi, 2018). Dendritic cells (DCs) are sensory cells and antigen-presenting cells (APCs) that have been found in RA synovial fluid (Thomas et al., 1999). In this study, we first established an *in vitro* DC inflammation model by stimulating mouse BMDCs with LPS, and we demonstrated that KPs can inhibit DC surface costimulatory molecule expression, reduce inflammatory cytokine release, and repress MAPK and NF-κB signaling. In a mouse model of CIA, we further observed that KPs can alleviate the symptoms of arthritis, decrease the production of inflammatory cytokines and suppress the maturation of splenic DCs.

The expression of the costimulatory molecules, including CD40, CD80 and CD86, is significantly increased in LPS-stimulated mouse BMDCs (Jones et al., 2010). We provided evidence that KPs treatment reduced the expression of these molecules in mBMDCs treated with LPS in a dose-dependent manner. In addition, LPS-primed mBMDCs were induced to secrete high-level cytokines, including TNF-α, a Th1-specific cytokine (IL-12), and Th17-specific cytokines (IL-6 and IL-23) (Siegemund et al., 2007). We demonstrated that KPs-pretreated mouse BMDCs secrete low concentrations of proinflammatory cytokines following LPS stimulation. Vinderola et al. (2005) showed that administration of kefir induces Th2 cytokine production to regulate the Th1 response in BALB/c mice. Two hundred milliliters of kefir were administered to 18 healthy subjects every day for 6 weeks to increase the polarization of the immune response toward a Th1-type phenotype and reduce the allergic reactions caused by the Th2-type response (Adiloğlu et al., 2013). In addition, a kefir product can activate the surface marker expression of human DC costimulatory molecules (CD80, CD86 and HLADR) and increase the release of cytokines (IL-1β, IL-6, IL-10 and TNF-α) (Ghoneum et al., 2015). However, our *in vitro* and *in vivo* experimental results showed that KPs inhibited the LPS-stimulated expression of BMDC costimulatory molecules and TNF-α, a Th1-specific cytokine (IL-12) and Th17-specific cytokines (IL-6 and IL-23). Therefore, we speculated that our KPs exert an inhibitory effect on DC maturation and cytokine production.

LPS treatment polarized CD4^+^ T cells and generated Th1 (IFN-γ) and Th17 (IL-17A) cytokine responses in previous studies (Jiang et al., 2002; McAleer and Vella, 2008; Fermin Lee et al., 2013). We analyzed the effects of KPs in a coculture system that included OVA-specific CD4^+^ T cells from OT-II transgenic mice and BMDCs subjected to different treatments. The results revealed that the proliferative responses were significantly reduced in LPS-stimulated DCs after KPs pretreatment. The results also showed decreased Th1 (IFN-γ) and Th17 (IL-17A) cytokine production in BMDC cocultures that had been treated with KPs.

Mounting evidence showed that several signaling pathway activation are involved in the DC maturation process (Neves et al., 2009). Especially the MAPK and NF-κB activation appeared to be extremely relevant to the process of LPS-induced BMDC maturation (An et al., 2002; Kim et al., 2005). In our study, we showed a similar result that three MAPKs (pERK, p-p38 and pJNK) and NF-κB signaling pathways were activated in LPS-induced DC (Figure 1). Kefir and its biological activities exerted anti-inflammatory and antioxidant effects through inhibiting p-p38 and p-NF-κB in several animal diseased model (Chen et al., 2020; Chen et al., 2019; Wang et al., 2012; Azizi et al., 2021) and exhibited antidepressant and antitumor effects through inactivation of p-ERK signaling pathway (Azizi et al., 2021; Chen et al., 2021). As anticipated, we showed a similar result as previous studies described, the levels of p-ERK and p-p38, and NF-κB/p65 nuclear translocation were significantly decreased under both doses of KPs treatment (Figure 1). However, inconsistent result was found in the level of p-JNK, which showed no significant change but slightly increased in KPs treatment when compared to LPS group. The results similar to the finding of Kim et al. (2021) showed that JNK phosphorylation increased in kefir-treated drug resistant cancer cells. Because the JNK signaling played a minor and distinct pathway from the p38, ERK, and NF-κB cascades in the LPS-induced DC maturation process (Nakahara et al., 2004). KPs still showed strong inhibitory effects on DC maturation and inflammatory cytokine releases in our study.

DCs have been found in RA synovial fluid, extensively expressing costimulatory molecules while performing their functions to polarize T cells toward Th1, Th2 or Th17 phenotypes. Therefore, DCs appear to play a crucial role in joint inflammation. In a typical arthritic animal model, CIA mice, the excessive production of pro-inflammatory cytokines or chemokines as well as continuous presentation of autoantigens by DCs are regarded as pathogenic factors, leading to the progression of murine arthritis. According to these findings, we investigated the role of KPs in the regulation of immune function in the CIA mouse model, which is used to study RA (Williams, 2004). We found that KPs treatment could suppress BMDC maturation and function; furthermore, KPs treatment for 3 weeks could also alleviate the symptoms of synovial tissue inflammatory infiltration, hyperplasia, and cartilage destruction in the knee joints of DBA/1 mice with CIA. Additionally, the ELISA results showed that the obvious expression of inflammatory cytokines, such as IFN-γ, IL-6, IL-17A and TNF-α, in the CIA/vehicle mice group was significantly reduced following high-dose KPs treatment (7.5 mg/kg/day). Therefore, KPs exert anti-inflammatory and immunomodulatory effects to prevent DC overreaction and mass accumulation in the synovial tissue of CIA mice.

Previous studies have revealed that berberine, an isoquinoline alkaloid, can be used to improve various autoimmune diseases by suppressing CII-specific immune responses in mice with CIA (Hu et al., 2011) or by suppressing Th17 cell responses via inducing cortistatin in the gut of rats with CIA (Yue et al., 2017). Accordingly, we further evaluated the production of anti-CII IgG antibodies and spleen cell proliferation responses in KPs-treated mice with CIA. We demonstrated that KPs treatment suppressed the anti-CII IgG antibody titers and spleen cell proliferation responses in mice with CIA. Similarly, the CD4^+^ T cell subset profiles in splenic lymphocytes were assessed. The ratios of Th1 and Th17 cells were also significantly decreased after oral administration of KPs in mice with CIA. It is well known that imbalanced Th17 cell production contributes to the progression of RA and CIA. Increased Th17 cell numbers were easily found in both the synovial fluid and peripheral blood of RA patients (Noack and Miossec, 2014). Our data demonstrated that KPs treatment is effective in lessening the ongoing severity of CIA in mice, and this effect involves the attenuation of the systemic Th17 cell response. In CII-treated spleen cells, KPs reduced the Th1 (CD4^+^IFN-γ) and Th17 (CD4^+^IL-17A) cell percentages in a dose-dependent manner. In our *in vitro* experiments, KPs inhibited the expression of costimulatory molecules in LPS-stimulated BMDCs. In an *in vivo* animal experiment, we also found that KPs inhibited the surface expression of CD40, CD80 and CD86 on splenic DCs in mice with CIA, which was consistent with a previous report (Jung et al., 2007). The abovementioned experimental results showed that KPs can inhibit the CII-specific T cell response. Based on the flow cytometry analysis, KPs treatment was sufficient to decrease the expression levels of these costimulatory molecules in an *in vivo* CIA mouse model and an *in vitro* LPS-induced BMDC cell model.

## Conclusion

In this study, these results demonstrated that KPs treatment can regulate immune functions to decrease inflammatory cytokine release, CD4^+^ T cell proliferation and Th1/Th17 cytokine production, to suppress splenic DC maturation and to alleviate all the symptoms of collagen type II-induced arthritis in the mouse model. Therefore, KPs treatment provide a novel perspective for investigating immune function in DCs and highlight the potential of KPs for the clinical use in RA patients.

## Data Availability

The original contributions presented in the study are included in the article/Supplementary Material, further inquiries can be directed to the corresponding authors.
