# Supplementary figures and images for: Suppression of Dendritic Cell Maturation by Kefir Peptides Alleviates Collagen-Induced Arthritis in Mice
Source: Front Pharmacol. 2021 Oct 5;12:721594. doi: 10.3389/fphar.2021.721594 (PMC8523924; doi:10.3389/fphar.2021.721594)

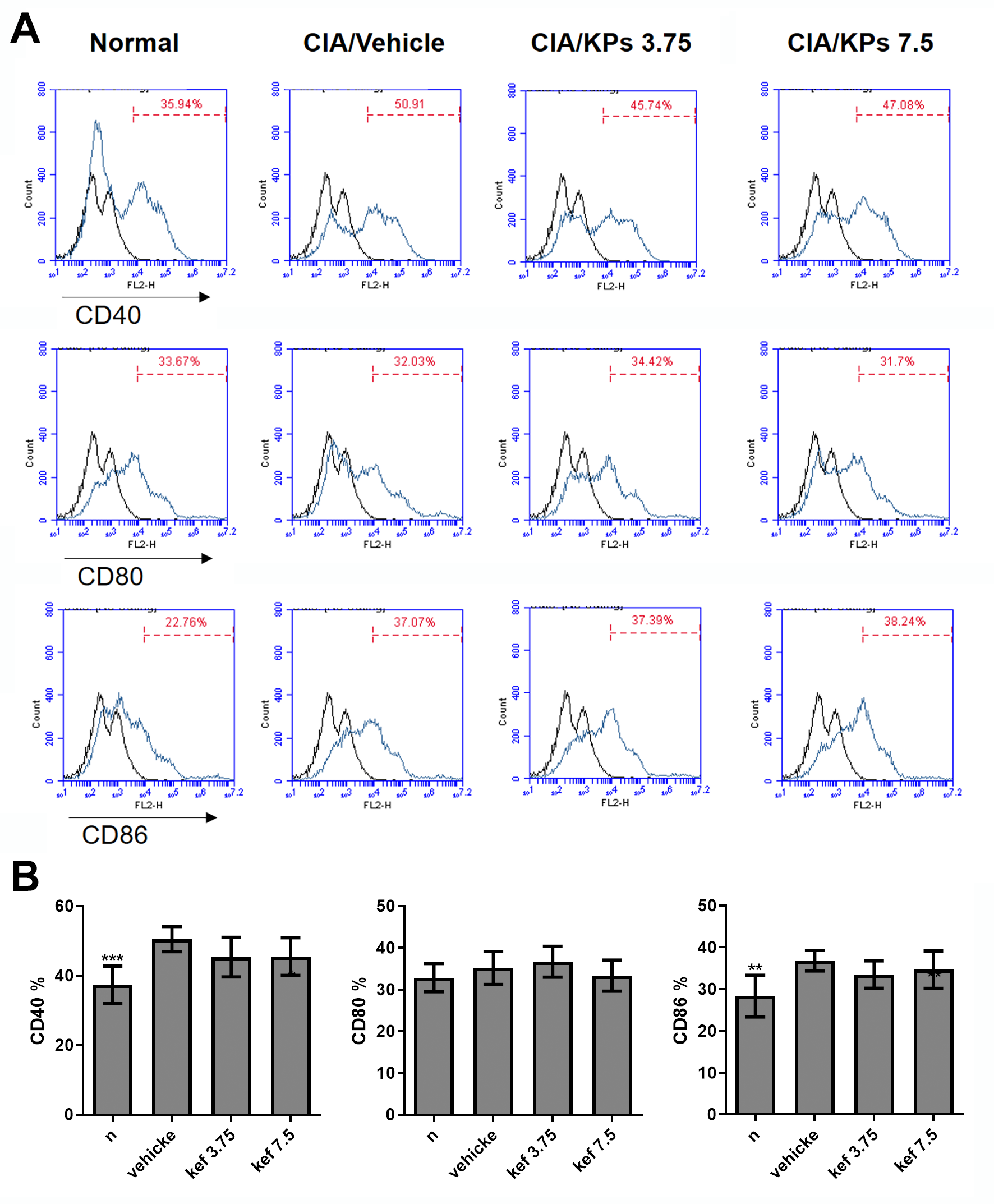

Supplement: Supplementary file 1 [file Image6.TIF]

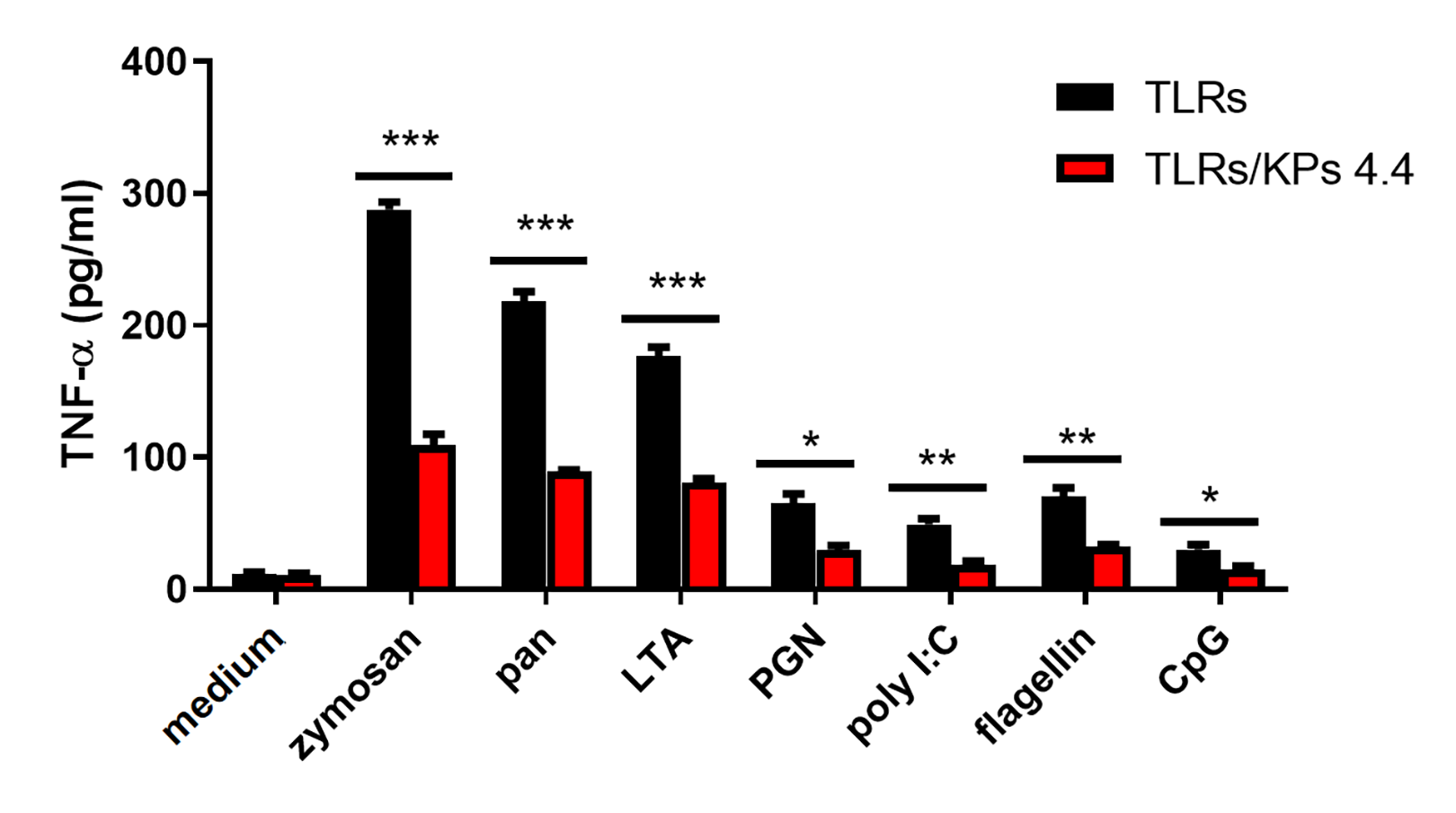

Supplement: Supplementary file 2 [file Image3.TIF]

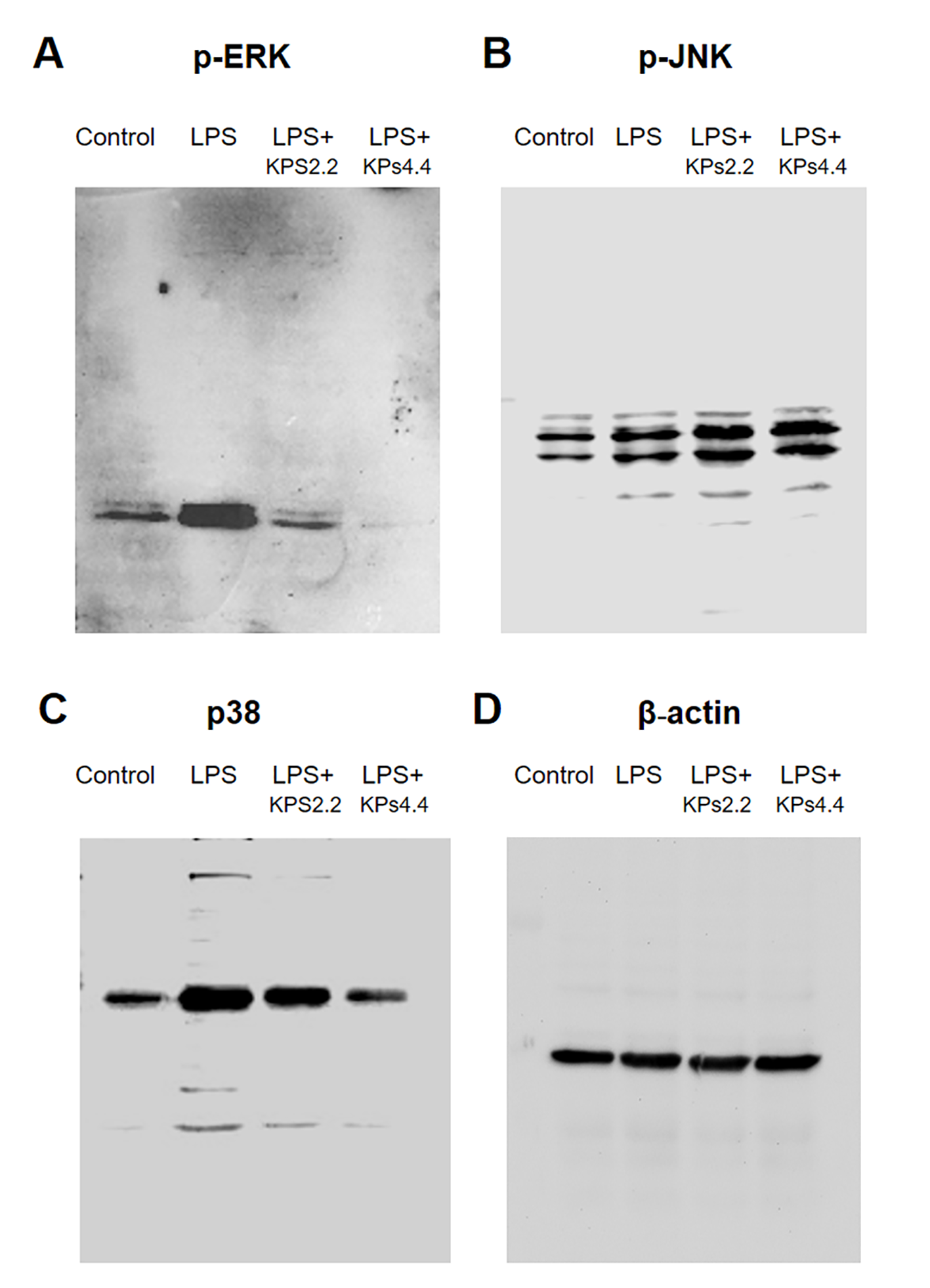

Supplement: Supplementary file 3 [file Image4.TIF]

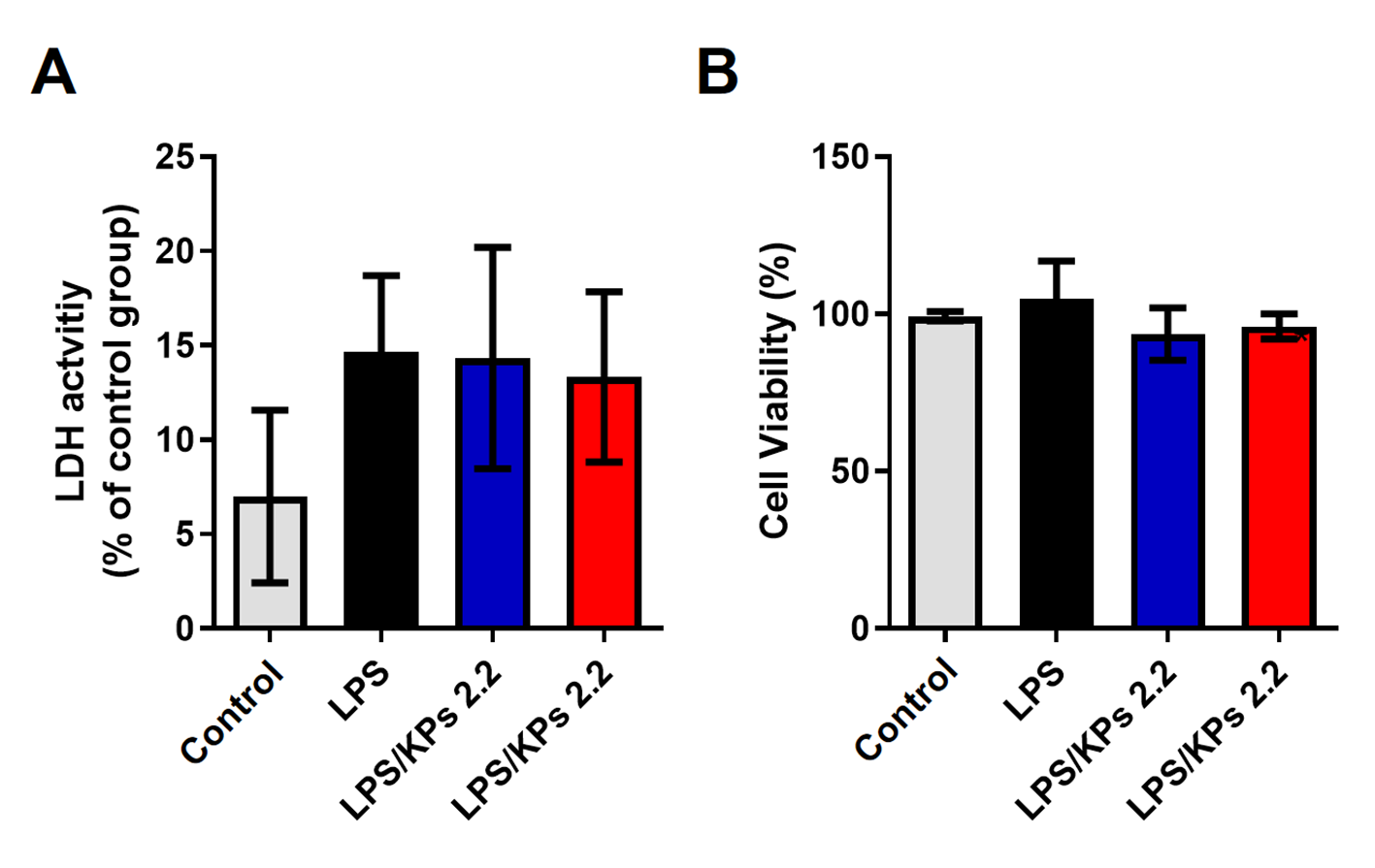

Supplement: Supplementary file 4 [file Image2.TIF]

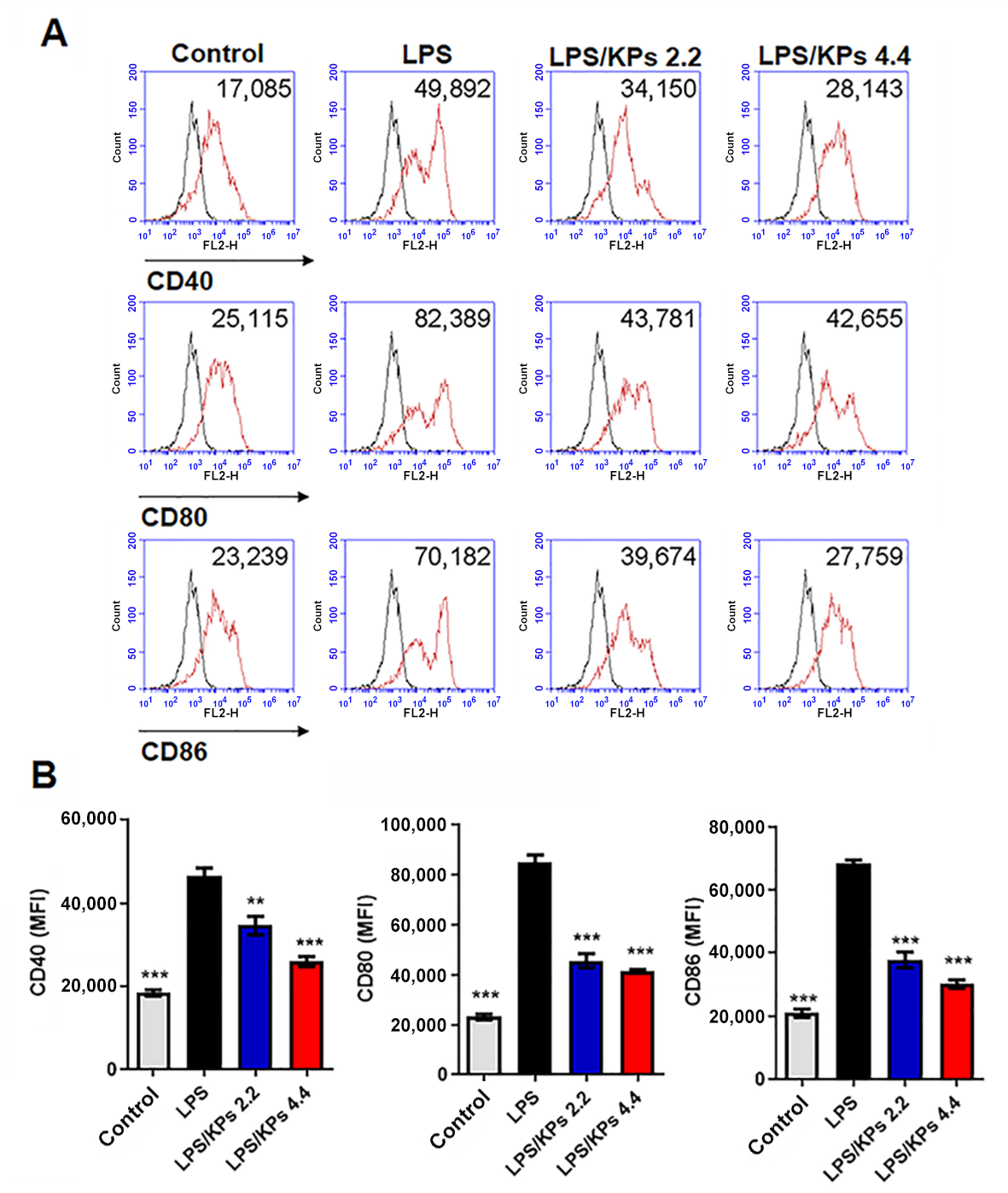

Supplement: Supplementary file 5 [file Image1.TIF]

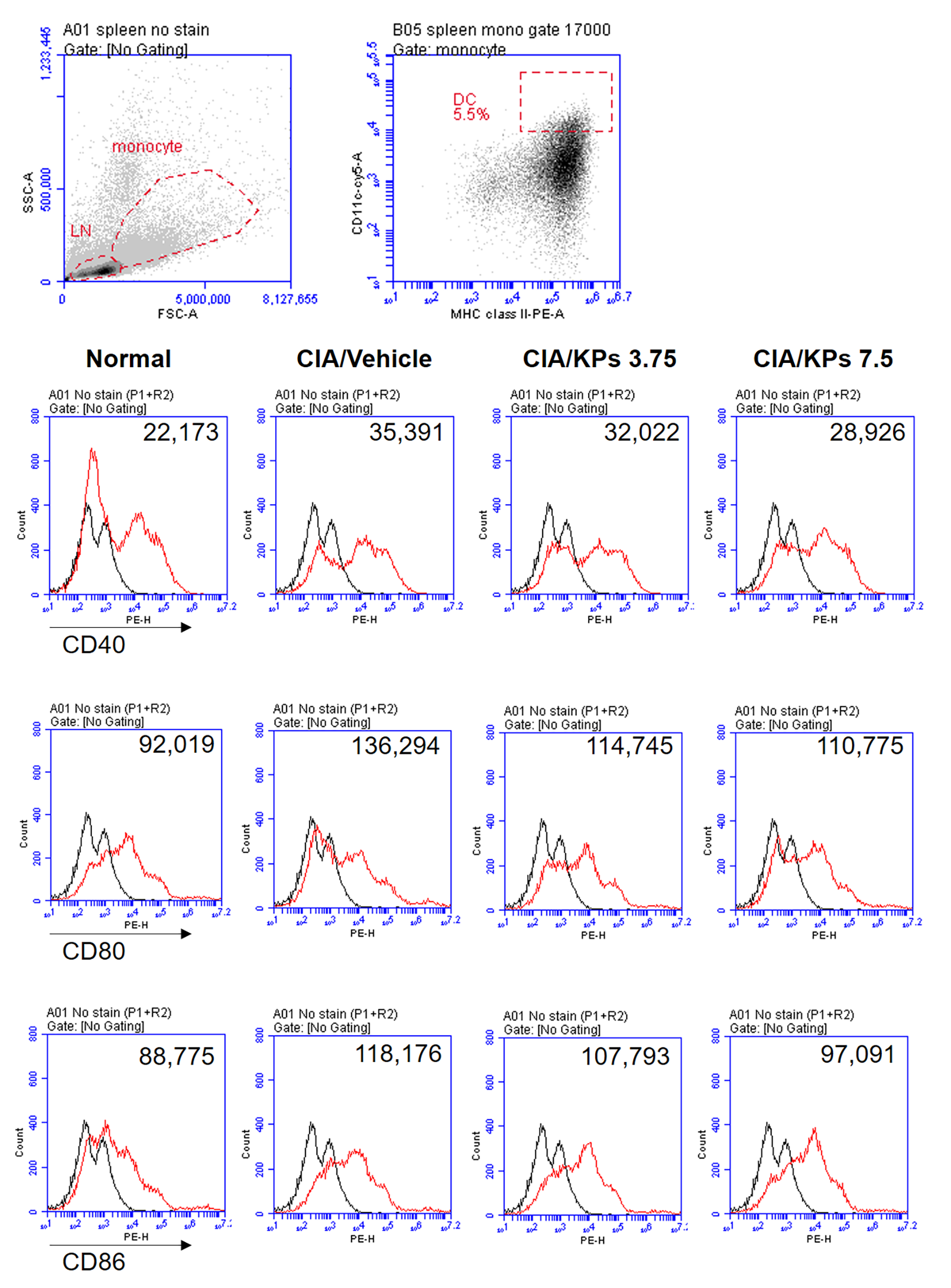

Supplement: Supplementary file 6 [file Image5.TIF]
